# Effectiveness of and Patient's Satisfaction with Dental Emergency Unit in Pitié Salpêtrière Hospital (Paris), Focusing on Pain and Anxiety

**DOI:** 10.1155/2022/8457608

**Published:** 2022-05-21

**Authors:** Grégoire Demeestere, Maxime Alcabes, Rafael Toledo, Isabelle Rodriguez, Yves Boucher

**Affiliations:** ^1^Université Paris Cité, Paris, France; ^2^Service d'Odontologie, AP-HP, Hôpital Pitié Salpêtrière, Paris 75013, France; ^3^Université Paris Cité, LabNOF, Paris 75006, France

## Abstract

**Background:**

The Dental Emergency Unit (DEU) of the Pitié Salpêtrière Hospital receives mainly painful emergencies. This study aimed at evaluating the suppression of pain and anxiety as well as the patient's satisfaction after a visit to the DEU. *Patients and Methods*. A prospective study was carried out in 2019 (NCT03819036) in adult patients. Data was collected on D0 on site and then on D1, D3, and D7 by phone, during daytime. The main objective and secondary objectives were, respectively, to assess the intensity of pain on D1; the intensity of pain on D3 and D7; the evolution of anxiety on D1, D3, and D7; and the patients' satisfaction. They were evaluated with a 0–10 numeric scale (NS) on D1, D3 and D7; mean scores were compared with nonparametric statistics (ANOVA, Dunn's test).

**Results:**

814 patients were contacted and 581 patients included; 87 were lost to follow-up. 376 patients completed all the questionnaires. In the final sample (59% men, 40 ± 16 y.o.), 86% had health insurance. The mean pain scores were as follows: D0: 6.36 ± 0.12; D1: 3.49 ± 0.13; D3: 2.23 ± 0.13; D7: 1.07 ± 0.11—indicating a significant decrease of 45%, 65%, and 93% on D1, D3, and D7, respectively, compared to D0 (*p* < 0.0001) between D0 and D1, D3, D7. The mean NS anxiety scores were as follows: D0: 3.32 ± 0.15; D1: 3.69 ± 0.16; D3: 2.75 ± 0.16; D7: 1.98 ± 0.15. The decrease was significant between D0 and D7 (*p* < 0.0001). The perception of general heath improved between D1 and D7. The overall score of satisfaction was 8.64 ± 0.06.

**Conclusion:**

DEU enabled a significant reduction in pain and anxiety with high overall satisfaction.

## 1. Introduction

Orofacial pain (OFP) is a very prevalent condition, estimated between 5% and 57% in adults in the last 12 months, depending on country, age group, and sociocultural level [1–13]. OFP is mainly caused by carious and periodontal diseases which are among the most prevalent diseases in the world [14, 15]. Tooth pain resulting from, for example, caries affects over 200 million worldwide and is the fifth most common acute condition observed overall [16]. The other most common sources of OFP are trauma, temporomandibular disorders [1, 17, 18], and other facial pains such as neuropathic pain [19]. OFP impairs biological, social, and psychological aspects of quality of life and oral health-related quality of life [20–24] with a high social and financial cost for individuals and society [25–27].

Even if some patients consult in the first place in medical services [28], OFP is mainly managed by dentists in a context of emergency within a framework of care specific to each country, being often private and public [29, 30]. The hospital is often considered a referral service for disadvantaged populations, for emergencies that exceed average competence, and for hours not covered by the main system [29, 31–39]. In France, emergencies are mostly regulated in private practices and hospitals, with permeability between the two systems [40]. Pain is the main reason for consultation in emergency settings [33, 41–44], and dental anxiety (DA), which affects about 20% of the population [45–51], is both an aggravating factor for pain and a factor delaying consultation [51–53].

There are however few studies devoted to the effectiveness of and satisfaction with emergency settings related to pain and anxiety [31, 43, 54–57]. The aim of this study was therefore to assess, using a prospective study, the effectiveness of the care provided in this emergency unit in terms of pain and anxiety, as well as measuring satisfaction, reflecting the patient experience. This study focused on data collected during daytime before COVID-19 pandemics; its main results have been published in French as preliminary data [58].

## 2. Patients and Methods

### 2.1. Study Design

The main objective of this study was to assess the reduction in pain 24 hours (D1) after the emergency visit (D0). The secondary objectives were to assess (1) the evolution of pain on D3 and D7; (2) the evolution of anxiety on D1, D3, and D7 after emergency visit; (3) the quality of reception of nursing and non-nursing staff; and (4) the perception of the quality of care.

A prospective monocentric observational cohort study was carried out from 01 April 2019 to 31 June 2019 at the Dental Emergency Unit (DEU) of the Pitié Salpêtrière Hospital (GHPS) in Paris. The study was approved by an IRB (APHP180366; IDRCB: 2018-A02692-53-CPP EST) and registered at clinicaltrials.org (NCT03819036). STROBE recommendations were followed for the design of the study and the writing of the report. Good Clinical Practices for clinical trials were supervised by the Clinical Research Unit (URC) of the GHPS.

### 2.2. Setting

The sample consisted of adult patients [>18 years old (y.o.)] presenting at the DEU of the GHPS for emergency care. The DEU is open 24 hours a day, 365 days a year, without phone call upstream regulating system. Care is provided by undergraduate students in their last year of study (6th year) under the responsibility of a senior (university hospital, hospital practitioner, or consultant). Upon arrival, patients take a ticket with a telephone number and time of arrival. They are called for care according to their order of arrival except for priorities (traumas, cellulitis, hemorrhages, pregnant women, children under 12 y.o., hospitalized or invalid patients, and prisoners). History taking, clinical examination and diagnosis are performed by students and controlled by a senior who validates the therapeutic decision, i.e., immediate care such as pulpotomy or avulsion, prescription, patient referral, one option not excluding the others. The treatment provided is the best suited to the situation of the patient and the service, as judged by the senior. For example, in case of AAA, the practitioner can either perform endodontic treatment or drainage in the service or prescribe antibiotic and analgesic treatment with referral to a GP. As the GHPS is an adult hospital, where children are only occasionally admitted, according to the state of emergency, the study was conducted in adult patients.

### 2.3. Diagnostic Criteria

Diagnostic criteria were defined according to international recommendations [59–63]: pulpal emergencies including reversible pulpitis (PR), irreversible pulpitis (PIR), acute apical periodontitis AP, acute apical abscess (AAA), periodontal abscess, pericoronitis, septum syndrome, ulcerative necrotic disease, alveolitis, cellulitis, temporomandibular disorder (TMD), oral mucosal pathology, and prosthodontics.

### 2.4. Participants

Participation in the study was proposed to all consecutive patients presenting in the DEU. Those satisfying eligibility criteria and willing to participate after receiving information were included after giving written consent. Inclusion criteria were age ≥18 y.o., patient available for phone calls during the week following the visit, and good understanding of the French language. Subjects with impaired communication were not included in the study.

### 2.5. Time Course of the Study

The study lasted 25 weeks and consisted of 3 one-week phases, with 2-month intervals, in order to limit selection bias. The maximum observation time of the patient was 10 days. Data collection was performed by 2 investigators (GD and MA). On D0 in the DEU, after inclusion in the study and medical history taking, the patients filled the sociodemographic and medical questionnaire and had clinical evaluation, diagnosis, and emergency care/prescription/advice according to their medical condition; before leaving, they filled the satisfaction questionnaire. On D1, D3, and D7, the evaluation questionnaires were completed by the investigators through a telephone interview.

### 2.6. Data Collection

Data were collected through questionnaires during the emergency visits and telephone calls. Sociodemographic data included social insurance status, habits of dental consultation, reason for consultation at the DEU, and how the patient attended the DEU. Medical data included medical history and actual treatments, diagnosis at the DEU, prescribed therapeutics, and further needed treatment. Efficacy measures included self-estimated pain and anxiety at the different time points of the study. Satisfaction measures included perception of the politeness of both medical and nonmedical staff, availability of nonmedical staff, care setting quality, cleanliness, waiting time, quality of medical information, and attitude toward recommending the DEU to friends.

### 2.7. Evaluation Criteria

The main evaluation criterion was the self-evaluated pain score assessed on a simple [0–10] numerical scale (NS) (0 = absence of pain; 10 = maximum pain score imaginable) [64], collected at baseline (D0) and on D1. The secondary evaluation criteria were (1) the self-evaluation of pain on D3 and D7, measured with the NS; (2) the score of anxiety on D0, D1, D3, and D7, measured on a [0–10] NS [65]; (3) the patient's perception of the quality of reception on D0, measured using a [0–10] NS; and (4) the evaluation of the quality of care assessed by the patient immediately after the treatment (D0), on a [0–10] NS.

### 2.8. Size Sample

We aimed at assessing 1% of the population consulting at the DEU. As a pilot study carried out in the service [66] showed that almost 50% of patients dropped out of the study after inclusion, with a number of consultations estimated at 44200 in 2017, the number of included patients was fixed at *N* = 800.

### 2.9. Data Analysis

The investigators were specifically trained for the phone interview. Patients not answering phone calls at the follow-up period were contacted the next day, and the pain and anxiety scores were extrapolated by calculating the nearest point on the curve connecting the previous and the newly collected data. If the patients were again not answering, the data was considered as missing. The data were anonymized throughout the study.

### 2.10. Statistical Analysis

A descriptive analysis of the sample was carried out. Pain and anxiety scores were grouped into 3 classes: absent or mild, from 0 to 4; moderate, from 5 to 7; and severe, from 8 to 10. Statistical analysis of the evolution of pain and anxiety scores on D0, D1, D3, and D7 was carried out with GraphPad Prism 5 software using ANOVA followed by Dunn's posttests. Correlations between the type of treatment and perceived pain as well as the type of treatment and perceived anxiety were searched with Pearson correlations (*R*^2^). Contingency analyses were performed with Chi^2^ test. Level of significance was set at 95%.

## 3. Results

### 3.1. Flowchart of the Study

**Figure 1:**
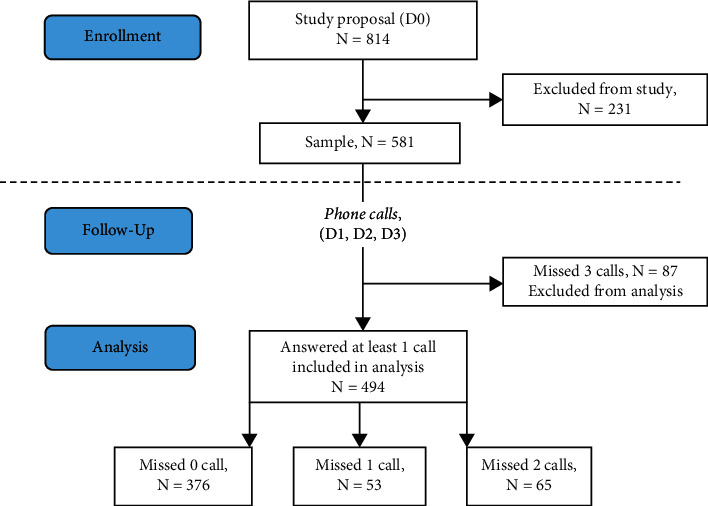
Flowchart of the study.

A total of 814 patients were contacted, of which 231 were excluded for different reasons according to inclusion and exclusion criteria (Figure 1). The final sample therefore included 581 patients. 376 patients answered all the calls, 53 missed one, 65 missed two, and 87 missed three. These 87 were considered lost to follow-up and not included in the analysis.

### 3.2. Characteristics of the Sample

#### 3.2.1. Sociodemographic

The sample included 59% men and 41% women, with an average age of 40 ± 16 years and a distribution of patients by age group as follows: <30: 30%; 30–39: 28%; 40–49: 16%; 50–59: 13%; 60–69: 7%; 70–79: 4%; ≥80: 2%. 14% of patients had no health insurance; 86% had it, including 69% social security (SS, classic health insurance), 10% CMU (universal health coverage, minimum coverage), and 6% AME (state medical aid). 74% declared no medical condition (MC) and 10% declared more than 2. Overall, the number of MC increased with age: patients with no MC represented 75% of the sample (mean age: 35.8 ± 0.6); patients with 1 MC represented 15% (mean age 49.8 ± 0.7); and patients with 2 MC or more represented 10% (mean age: 56.8 ± 0.9). 58% of the sample reported a visit to the dentist less than 1 year ago. However, 25% had none for more than 2 years, including 15% with more than 5 years. Overall, dental follow-up increased with the quality of health insurance; patients benefiting from dental follow-up of less than one year were indeed 35% without health insurance, 41% with AME, and approximately 60% with CMU and SS. Conversely, the decrease in social protection tended to space follow-up visits: 33% of patients without SS, 18% with AME, 16% with CMU, and 11% with SS had the last dental follow-up more than 5 years ago. There was a statistically significant difference in pain scores between subjects having a regular SS (6.14 ± 0.15 vs. 6.88 ± 0.21; *p* < 0.001) and those with no or minimum health insurance. 61% of the sample came directly to the DEU while 39% tried to have an appointment with a dentist before. Among the latter, 17% were addressed by the contacted dentist, 15% could not wait until the proposed appointment, and 7% evoked other reasons such as the cost of the care in a private practice. including financial reasons (1%). Patients came to the GHPS on the advice of relatives (27%), because they had already come (20%), after Internet search (14%), already informed by a dentist (12%), and 26% knew the DEU by other means.

#### 3.2.2. Clinical Characteristics of the Sample

The main reason for consultation was pain (91.6%) including pain only (71.7%) or associated with either swelling (15.9%), mobility (1.9%), or trauma (1.2%). Among the 8% with no pain, 3.2% consulted for seeking advice.

The medical diagnoses sorted by frequency were AAA (acute alveolar abscess, 19.9%), PAA (acute apical periodontitis, 19.2%), irreversible pulpitis (12.5%), cellulitis (9.2%), pericoronitis (5.8%), periodontal abscess (4.6%), trauma (3%), reversible pulpitis (2.1%), and septum syndrome (1.3%). The rest (2%) were pathologies of the oral mucosa, salivary pathologies, patient orientation errors, etc.

### 3.3. Efficacy

#### 3.3.1. Pain

**Figure 2:**
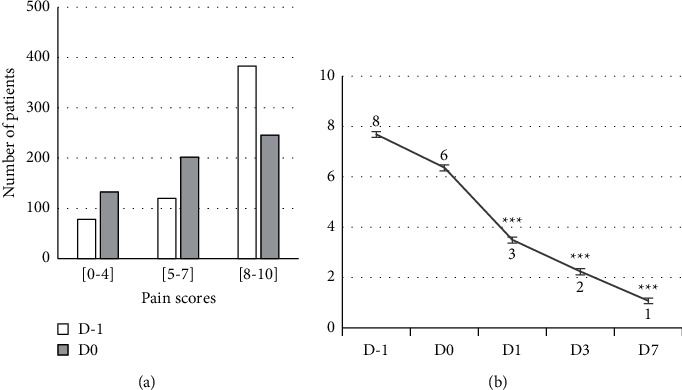
(a) Distribution and evolution of pain scores on D1 and D0 according to severity of the pain. (b) Evolution of pain scores (mean ± SEM) from D1 to D7 indicating a significant decrease between baseline (D0) and endpoints (D1, D3, D7) (ANOVA, Dunn's test, *p* < 0.001). Pain scores were self-evaluated on a [0–10] numeric rating scale.

The distribution of pain scores on D1 and D0 and their evolution from D1 to D7 are illustrated in Figure 2. Mild and moderate pain were more frequent on D0 whereas an opposite trend was observed for severe pain. Mean pain scores on D1, D0, D1, D3, and D7 were, respectively, 7.68 ± 0.12, 6.36 ± 0.12, 3.49 ± 0.13, 2.24 ± 0.13, and 1.07 ± 0.13, i.e., a respective suppression of 45%, 65%, and 83% on D1, D3, and D7. This diminution was statistically significant at all endpoints (ANOVA, Dunn's test, *p* < 0.001). An already significant decrease is observed between D1 and D0.

**Figure 3:**
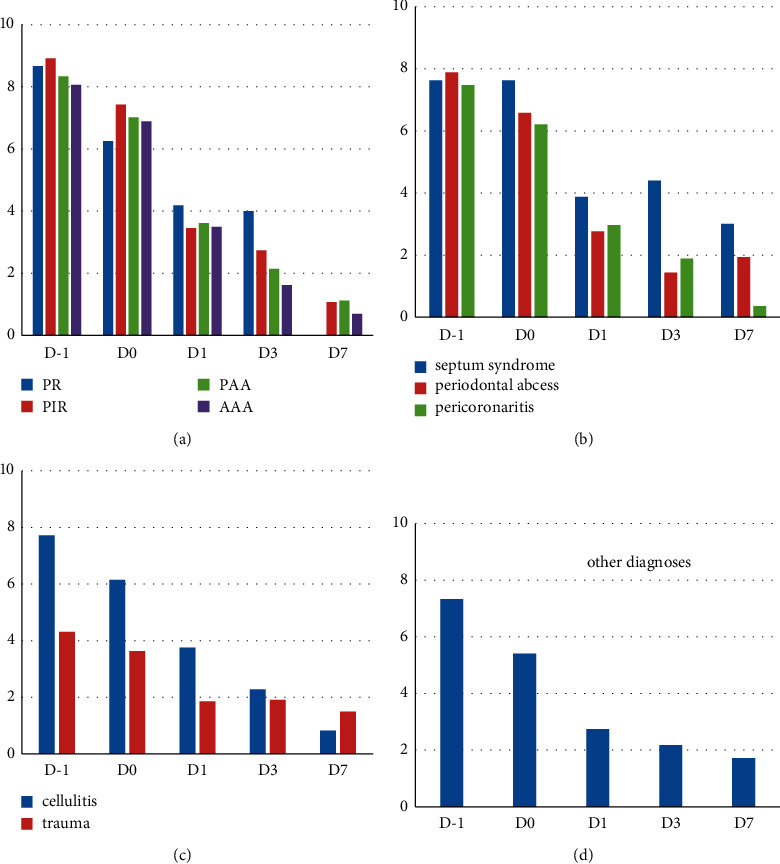
Distribution of self-evaluated [0–10] pain scores (ordinates) on D1–D7 (abscissae) according to diagnosis. (a) Pulpal pain (AAA, PAAA, PIR, PR). (b) Periodontal pain (septum, periodontal abcess, pericoronitis). (c) Cellulitis and traumas. (d) Other diagnoses including TMD, oral mucosal pathologies, and prosthodontics. Note. PR: reversible pulpitis, PIR: irreversible pulpitis, AP: acute apical periodontitis, AAA: acute apical abscess.

Pain scores and distribution according to diagnoses are illustrated in Figure 3. Pain motivating the visit was mainly of pulpal origin (54.5%) distributed as follows: AAA (20.2%); APP (19.5%); PIR (12.7%); PR (2%); and other diagnoses (45.5%): cellulitis (9.4%), periodontal abcess (4.5%), pericoronitis (5.9%), traumas (3.3%), and septum syndrome (1.4%). Scores for all categories decreased significantly between D0, D1, D3, and D7 (ANOVA, *p* < 0.001), except for traumas.

#### 3.3.2. Patients Not Answering Phone Calls

**Figure 4:**
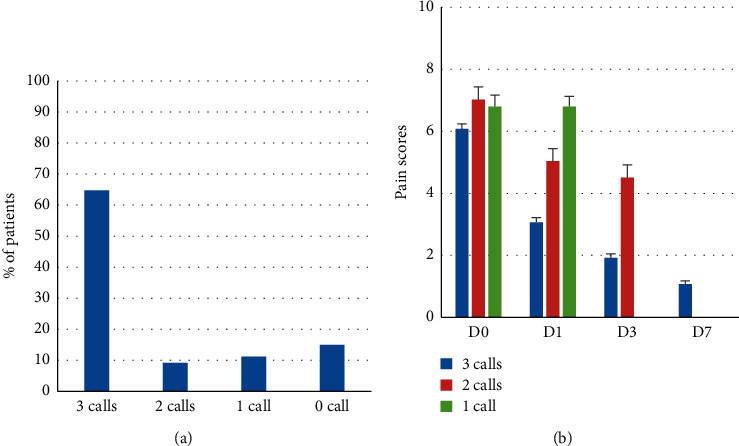
Pain status of responders and nonresponders to phone calls. (a) Percentage of patients responding to phone calls. (b) Pain score according to the number of calls.

15% of the sample (*N* = 87) did not answer any call and were considered as non-responders (NR) and excluded from further analysis, while 85% (*N* = 494) answered at least one call. Mean pain scores on D0 were not significantly different for NR (6.9 ± 0.28) and responders (6.3 ± 0.09) (Mann & Whitney *p*=0.32). In the subgroup of responding patients, 376 (65%) responded to the 3 recalls, 53 (9%) to 2, and 65 (11%) to 1 with a D0 respective pain score of 6.1 ± 0.16, 7.0 ± 0.41, and 6.8 ± 0.36. There was a significant difference in pain scores on D1 for patients responding to 1, 2, or 3 calls; the more the patients responded, the more the pain decreased (Figure 4).

#### 3.3.3. Anxiety

**Figure 5:**
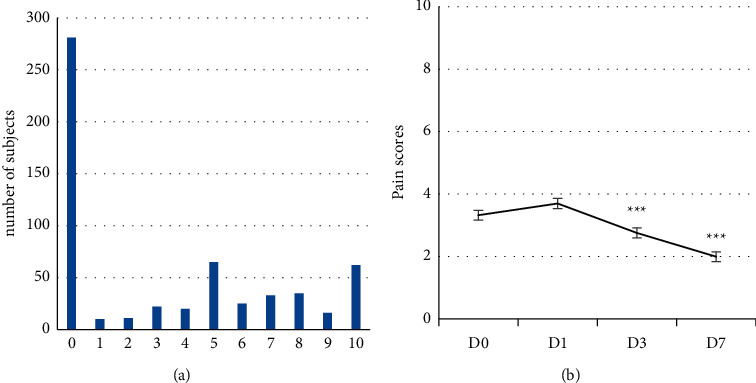
Anxiety. (a) Distribution of anxiety scores on D0, self-evaluated on a [0–10] numeric rating scale. (b) Evolution of anxiety scores from D0 to D7. A significant decrease is observed between baseline on D3 and D7 (ANOVA, Dunn's test, *p* < 0.001).

The overall level of anxiety on D0 was low since 57% of the scores were in the range 0–4, including 49% with no anxiety and 20% with moderate anxiety (scores: 5–7) (Figure 5). Severe anxiety (scores: 8–10) represented 23% of the sample. The mean anxiety scores were as follows: D0: 3.32 ± 0.15; D1: 3.69 ± 0.16; D3: 2.75 ± 0.16; D7: 1.98 ± 0.15. The decrease was significant between D0 and D7 (ANOVA, Dunn's test, *p* < 0.001). On D7, low scores of anxiety constituted 79% of the sample, moderate scores constituted 12%, and high scores constituted 9%.

#### 3.3.4. General Health

Overall, the perception of the patient's general health (GH) improved significantly with time. The average GH scores increased significantly (ANOVA, *p* < 0.001) from 7.56 ± 0.09 on D1 to 7.95 ± 0.10 on D3 (*p* < 0.05) and 8.25 ± 0.10 on D7 (Dunn's test,*p* < 0.001).

### 3.4. Treatments and Further Attendings

52% of the patients received a medical prescription alone while 8% received only advice without treatment or prescription. 21% of patients benefited from surgical treatment (extraction), and 18% benefited from endodontic treatment. Level of pain was slightly correlated with the type of therapeutic response; i.e., higher levels of pain resulted more often in surgical/endodontic treatment than lower ones (Chi^2^, *p*=0.02).

In the sample, 9%, 12%, and 19% (*N* = 70) of the patients consulted again on, respectively, D1, D3, and D7. The mean reasons for reconsulting were persistent pain (30%) and worsening of the swelling (12.9%) while others came for extraction (24.3%) or control (32.9%). Observance of the instructions given on D0 was reported by 96% of the patients on D1 and 93% on D7, mainly because the patients stopped antibiotics with the subsiding of the pain. 60.2% of patients made an appointment for further care on D1 with a dentist, either in a private practice or at the GHPS, 68.5 on D3, and 70.3% on D7.

### 3.5. Socioeconomic Characteristics, Pain, and Anxiety

No correlation was found between pain and anxiety scores (*R*^2^ = −0.09; *p*=0.8). Women reported significantly higher scores of pain than men (Chi^2^, *p* < 0.001), although mean pain scores were not significantly different: 6.49 ± 0.19 vs. 6.27 ± 0.17. There was a statistically significant difference between pain scores according to health insurance status with mean scores of 6.14 ± 0.15 vs. 6.85 ± 0.21 (*p* < 0.05) for, respectively, patients with SS and patients with minimum health coverage (AME, CMU, no insurance), although no statistically significant difference between subcategories could be found (ANOVA, *p*=0.3). The difference in pain scores between patients who consulted a dentist during the past year, 1-2 years ago, 2–5 years ago, and >5 years ago was not significant: 6.1 ± 0.17, 6.8 ± 0.26, 6.7 ± 0.39, and 6.5 ± 0.33, respectively.

### 3.6. Satisfaction

**Figure 6:**
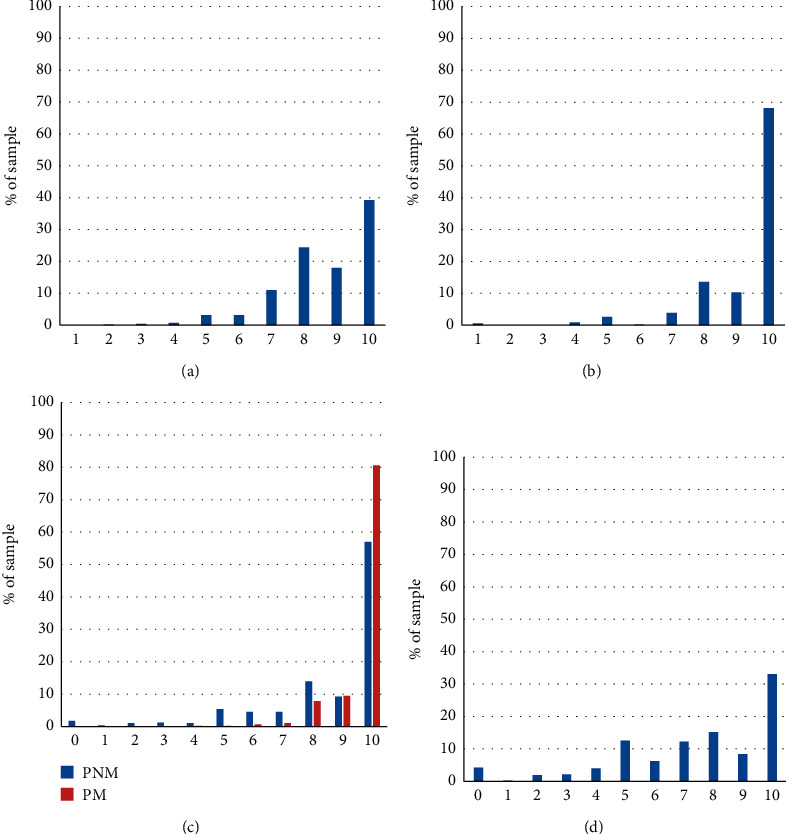
Evaluation of satisfaction. (a) Distribution of overall satisfaction scores on D0 self-evaluated on a [0–10] scale (abscissae) (b) Distribution of scores of perception of quality of medical treatment and information. (c) Distribution of scores of politeness and availability of medical (PM) and nonmedical (PNM) staff. (d) Perception of waiting time in the sample.

The average overall satisfaction score after visit to the DEU was 8.6 ± 0.06 (Figure 6(a)). Perception of the quality of medical care was high (mean score: 9.3 ± 0.05) (Figure 6(b)). Mean score for politeness and availability of nonmedical staff and medical staff was, respectively, 8.6 ± 0.09 and 9.6 ± 0.03 (Figure 6(c)). Almost all patients (97.2%) were satisfied with the information received in the DEU, including postoperative instructions. The average total time spent in the DEU including the waiting room, consultation, and treatment, was 2.24 h ± 0.05 ranging from 0.5 h to 7 h). Mean score of satisfaction regarding waiting time was 7.4 ± 0.11. Waiting time was the main source of dissatisfaction with 12.3% of the sample rating their satisfaction below average (5/10). There was a positive correlation (Pearson *R*^2^ = 0.613, *p* < 0.0001) between the overall score of satisfaction and the waiting time. The overall satisfaction was confirmed by the high percentage of patients intending to recommend the DEU to a friend/family member or consult again (98%).

## 4. Discussion

The main results of this study are a high prevalence of pain and anxiety in the population consulting the DEU and a high efficiency of the DEU in reducing both of them, measured at 3 endpoints. Pain decreased on average by 83% and anxiety by 40% between D0 and D7. Furthermore, the perceived satisfaction was excellent since 97.8% of the sample gave an overall satisfaction score of ≥5 with a mean score of 8.6/10.

### 4.1. Sociodemographic Characteristics of the Sample

There are relatively few cohort studies related to dental emergencies which overall tend to indicate that patients attending emergency departments are rather young and male with low socio-medico-economic status [5, 7, 31, 33, 42, 43, 56, 66–70], therefore consulting dentists less regularly than general population. DEU is often considered as an alternative to regular follow-up for patients with low access to dental care. However, in the present study, only 31% of the patients had either no healthcare insurance or minimal coverage (AME, CMUs), indicating that not only is the DEU a recourse for socioeconomically disadvantaged individuals; the DEU of the GHPS might also be considered more as a first line setting than as a recourse setting since only 6.7% of patients were examined and referred by a dentist before coming to the DEU, although less than 15% of the emergencies were life-threatening conditions (cellulitis, hemorrhages) or traumas. This first-visit rate is high and comparable to [70, 71] where 80% and 56% of patients were found to directly attend the DEU. As in France 86% of the dentists work in private offices, 12% are employed in private centers or company/association settings, and only 2% work in public hospitals [72], this suggests an inability of the actual health system to adequately respond to dental emergencies. The reasons of these findings have to be explored, but this does not result from an underpopulation of practitioners since the Paris area is the densest of the country; it might result from a reluctance of private practitioners to admit new patients in emergency because of either the disorganization effect on working schedule and economically/technically ungratifying type of care or a specific reluctance to receive patients with low socioeconomic status [73]. Whatever the reason is, this is worrying with regard to the consequences for pain and anxiety in the population in terms of individual quality of life and socioeconomic costs. In the sample, 42% of the patients did not consult in the past year and 15% during the past 5 years, which confirms and updates previous national data [74]; patients with regular control consult statistically less in emergency settings than others, with the exception of traumas [31, 32, 75]. In this study, patients having consulted in the elapsed year had significantly lower pain scores than others. However they constituted the majority of the sample (58%).

### 4.2. Reasons for Consultations, Diagnostics, and Efficacy of the DEU

#### 4.2.1. Pain

Pain was the main reason for consultation in 91% of the sample, being associated with swelling in 16% or trauma in 2%, which is in the upper range of emergency studies [39%–88%] [31, 42, 43, 56, 68, 76–78]. Pain was mainly of infectious origin (75.7%) including 54.5% pulpal pain (PR, PIR, PAA, AAP), 11.8% periodontal/mucosal pain (septum syndrome, periodontal abcess, pericoronitis), and 9.4% cellulitis regardless of its origin. These infections are the most prevalent worldwide and could be often prevented with regular visits and adequate prevention programs. Severe pain, i.e., scores [8–10], on D0 constituted 42.3% of the sample. Pain scores measured at baseline and at 3 endpoints were high on D0 (6.364 ± 0.12) and low on D7 (1.07 ± 0.13) with an almost linear decrease suggesting that consultations were effective regarding pain control. Only 21% of the patients had a surgical procedure performed in the DEU (avulsion, endodontic treatment, or surgical drainage) which could be accountable for the rapid decrease of pain; medication, medical advice, empathy, or natural course of pain also might have contributed to the pain suppression. For example, pain intensity was higher the day before the consultation for severe pain, and it was the opposite for low and moderate pain. This could reflect the patient expectations [79], positive contextual effect of the care [80], or natural evolution of the disease, for example, natural drainage of an abcess or necrosis of the pulp evolving into an asymptomatic state. Whatever the reason is, the DEU was beneficial to these patients although it is important to keep in mind that on D7, 8.8% of the patients still experienced a moderate to severe level of pain, scores [6–10]. Many of the patients could have attended a regular dental office since less than 15% had a medical threatening condition or trauma; however, 42% had a severe pain which can be considered a medical emergency. The present results must also be weighted according to the status of respondents/nonrespondents. The analysis was performed on respondents, i.e., those who answered at least one call on either D1, D3, or D7, which can be a bias since nonrespondents might be less likely to answer calls when the treatments were not considered as satisfactory. Indeed, a significant difference was found between levels of pain of respondents and nonresponders on D1, D3, and D7. Overall, these data emphasize the important role played by dentists in the control of pain, which becomes more important with prevention programs.

#### 4.2.2. Anxiety

Anxiety scores on D0 were low (3.3 ± 0.15), although not uniformly distributed since 30% reported scores of [5–10] and 20% reported scores of [8–10], which can be compared with other studies reporting similar findings with different evaluation tools [42, 81]. The consultation had a significant effect on anxiety which decreased to 26% on D3 and 43% on D7 compared to D0. This reduction of anxiety is beneficial for quality of life [82]. Indeed, it was accompanied by a significant improvement of the perception of the general health on D3 and D7. The majority of the patients declared compliance with prescription, and 9 patients out of 10 did not consult again between D0 and D7, suggesting again that emergency treatment was beneficial.

#### 4.2.3. Satisfaction

In the present study, the overall perception of the DEU was excellent, reflecting specific satisfaction with quality of care and attitudes of both medical and nonmedical staff. The worse item was waiting time which exceeded 4 h in 7.8% of the sample. Few studies precisely assessed waiting time in DEU, which is part of patient satisfaction [78], especially in a context of pain and anxiety [83]. In the present study, there was a significant correlation between waiting time and overall satisfaction. It should be noted that satisfaction was not only the reflection of pain relief since the evaluation was performed immediately after the visit on D0, when medication and procedures were sometimes not fully effective in pain reduction. However, among the many medical and nonmedical parameters contributing to patient satisfaction [84], pain relief remains the main expectation of the patient, independently of age, sex, or intensity of pain [84–86], and pain relief is correlated to patient expectations [79]. Unfortunately, no reevaluation of satisfaction on D1, D3, or D7 was performed in this study.

#### 4.2.4. Care Provided

Care provided in the DEU is the most adapted to condition of the patient according to the resources of the service. In the present sample, 52% of the patients received a prescription, 21% had extraction, 18% got endodontic treatment (pulpotomy/pulpectomy) or cleaning/disinfection of infected root canals, and 8% received solely advice. The specific nature of the treatment is of little importance for the patient, who is more concerned with the relief of pain and attitude of caregiver which includes empathy, listening, explanations, and advice, especially in a context of emergency [35, 80].

### 4.3. Strengths and Limitations

#### 4.3.1. Strengths

The results of this study are very similar to the pilot study of [66] with a few socioeconomic differences mainly related to (1) the distribution of the health insurance affiliations, (2) the reason for consultation with a 16% increase for pain, and (3) the consultation of a dentist during the past year which increased by 17%. Regarding medical data, the overall distribution of diagnoses, scores of pain and anxiety, and measures of efficacy and perception of general health was also very similar. A one-point increase in the overall satisfaction score was also noted. The comparability increases the value of the present study. It can be noted that the GHPS is one of the well-known hospitals in France and is easily accessible with public transportation.

#### 4.3.2. Limitations

A potential selection bias is related to language. The patients who do not speak French enough to understand the protocol and questionnaires have been excluded. They might belong to a lower socioeconomic status and health coverage. This study evaluated the DUE only during working days and daytime. Out-of-hours periods have been evaluated in an ancillary study [87]. The same study has also been performed during COVID-19 time [88].

This study focused on daytime emergencies and did not evaluate out-of-hours periods. Therefore, an ancillary study was planned (NCT04352881) but interrupted by the COVID-19 pandemics. During COVID-19 time, an additional study (NCT04354272) performed with the same methodology was launched in order to compare pain anxiety and efficacy of the DEU during the two periods. These two ancillary studies will complete the present data when published.

## 5. Conclusion

Measuring the quality of care is a complex issue including many domains and dimensions [89]. This study documents the efficacy of the DEU in suppressing pain and anxiety. It suggests that the DEU has both medical and social utility.

## Data Availability

Data are available from the corresponding author on demand.
